# Late-phase miRNA-controlled oncolytic adenovirus for selective killing of cancer cells

**DOI:** 10.18632/oncotarget.3350

**Published:** 2015-01-31

**Authors:** Xavier Bofill-De Ros, Eneko Villanueva, Cristina Fillat

**Affiliations:** ^1^ Institut d'Investigacions Biomèdiques August Pi i Sunyer (IDIBAPS), Barcelona, Spain; ^2^ Centro de Investigación Biomédica en Red de Enfermedades Raras (CIBERER), Barcelona, Spain

**Keywords:** Oncolytic adenovirus, fiber protein, L5 gene, miRNAs, pancreatic cancer

## Abstract

Tissue-specific detargeting by miRNAs has been demonstrated to be a potent strategy to restrict adenoviral replication to cancer cells. These studies have generated adenoviruses with miRNA target sites placed in the 3′UTR of early gene products. In this work, we have studied the feasibility of providing tissue-specific selectivity to replication-competent adenoviruses through the regulation of the late structural protein fiber (L5 gene). We have engineered a 3′UTR containing eight miR-148a binding sites downstream the L5 coding sequence (Ad-L5-8miR148aT). We present *in vitro* and *in vivo* evidences of Ad-L5-8miR148aT miRNA-dependent regulation. *In vitro* data show that at 72 hours post-infection miR-148a-regulation impaired fiber expression leading to a 70% reduction of viral release. The application of seven consecutive rounds of infection in miR-148a cells resulted in 10.000-fold reduction of viral genomes released. *In vivo*, liver production of infective viral particles was highly impaired, similarly to that triggered by an adenovirus with miRNA target sites regulating the early E1A gene. Noticeably, mice treated with Ad-L5-8miR148aT showed an attenuation of adenoviral-induced hepatotoxicity but retained full lytic activity in cancer cells and exhibited robust antitumoral responses in patient-derived xenografts. Thus, miRNA-control of late proteins constitutes a novel strategy to provide selectivity to adenoviruses.

## INTRODUCTION

Oncolytic adenoviruses hold great promise for cancer treatment since they can replicate and destroy cancer cells. However, the antitumor efficacy observed in clinical trials has been modest. The development of more potent oncolytic adenoviruses faces with a potential increase in associated-toxicities. Systemic administration is necessary for the treatment of metastatic disease, however the intravenous administration of adenovirus results in viral retention in the liver causing significant infection of hepatocytes leading to liver damage [1]. To improve the safety of oncolytic adenoviruses a control of the viral tropism based on tissue-specific miRNA regulation has been exploited. MicroRNAs (miRNAs) are small noncoding RNA molecules with important regulatory roles in gene expression. They act by binding to the 3′-untranslated region (3′UTR) of targeted messenger RNAs (mRNAs), promoting either mRNA cleavage or repression of gene expression at post-transcriptional level [2]. Nowadays, miRNA deregulation is considered a hallmark in cancer with several miRNAs described to be downregulated in a variety of tumors [3]. Since low expression of miRNAs with respect to normal tissue represents a common trait in some neoplastic cells, this has been exploited as a mechanism to control the adenoviral tropism [4]. miRNA-122 recognition of engineered target sites in the 3′UTR of the E1A gene to control its expression and prevent viral replication of the adenovirus has been the most widely exploited strategy since miR-122 is abundantly expressed in human and murine liver [5, 6]. Substantial decrease in viral replication has been reported in 4 or 8 engineered target sites for miR-122, with highly reduced hepatotoxicity [7]. We have recently shown that the inclusion of 8 target sites downstream E1A recognizing miR-148a/miR-152 family members efficiently detargeted adenovirus from mouse liver and normal pancreas while maintained its antitumoral activity in pancreatic tumors [8].

In the current work, we explored whether targeting the mRNA of structural components of the virus, expressed at the late phase of the adenoviral life cycle, would be a feasible strategy to control the adenoviral tropism. The majority of Ad5 late proteins, from L1 to L5 are encoded within the Major Late Transcription which is driven by the Major Late Promoter and are defined by distinct poly(A) sites [9]. The complexity of MLP regulation is not compatible to provide adenovirus with tumor selectivity through the use of tumor-specific promoters, a strategy widely studied to drive E1A. Thus, we hypothesized that the insertion of miRNA-target sites controlling L5 could provide a novel region in the adenoviral genome to be engineered to control viral replication in a tumor-selective manner.

Our data shows that miR-148a attenuates the expression of the fiber protein in cells or tissues infected with the Ad-L5-8miR148aT engineered virus. Furthermore, adenoviral replication of Ad-L5miR148aT is strongly impaired both, *in vitro* and *in vivo*, in miR-148a expressing cells and tissues, but it retained full efficacy against pancreatic xenografts. Toxicity effects associated to liver damage at earlier time points were slightly ameliorated. This work provides compelling evidences that miRNA regulation of gene expression and viral replication can be adapted to the control of structural proteins of the adenovirus.

## RESULTS

### MiR-148a reduces fiber gene expression and decreases Ad-L5-8miR148aT viral production

**Figure 1:**
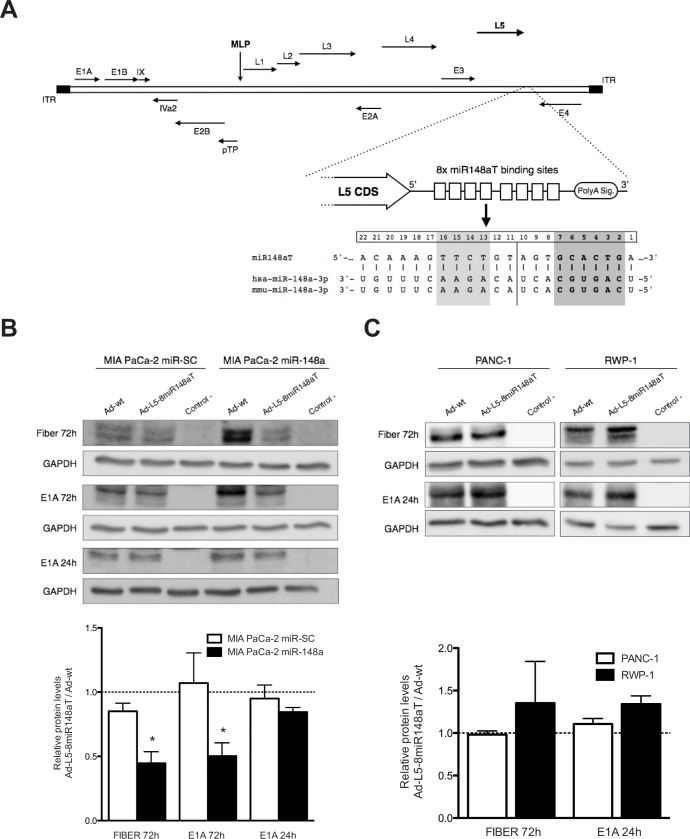
miR-148a regulates fiber expression from cells infected with Ad-L5-8miR148aT (A). Scheme of miRNA target sites engineered in the L5 viral gene. B, C. (Upper panels) Representative Western blots of E1A and Fiber from (B) MIA PaCa-2 miR-148a and MIA PaCa-2 miR-SC or (C) PANC-1 and RWP-1 cells infected with 50 vp/cell of Ad-wt and Ad-L5-8miR148aT for 24 and 72h. (Lower panels) Histograms representing the densitometric analysis of Fiber and E1A signals, normalized against GAPDH and expressed as relative to Fiber and E1A of Ad-wt infected cultures. Data is shown as the mean ± SEM of four independent experiments.

**Figure 2:**
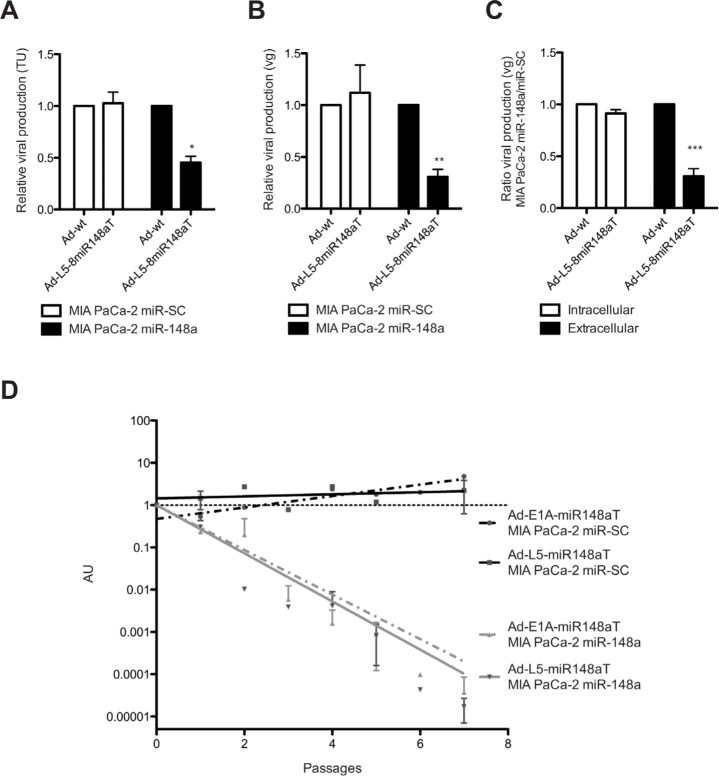
miR-148a regulates viral release from cells infected with Ad-L5-8miR148aT A, B. Quantification of viral production from supernatants obtained from cells infected with 50 vp/cell of Ad-wt and Ad-L5-8miR148aT at 72h post-infection by (A) hexon immunostaining, (B) qPCR. Data is shown as the mean ± SEM of five (A) and four (B) independent experiments. ** p<0.01 and *** p<0.001. (C). Quantification of viral production in supernatant (extracellular) and pellets (intracellular) of cells infected with 50 vp/cell of Ad-wt and Ad-L5-8miR148aT at 48h post-infection by qPCR. Data is shown as the mean ± SEM of three independent experiments. * p<0.05. (D). Quantification of viral production in supernatant of cells infected with Ad-wt, Ad-E1A-8miR148aT and Ad-L5-8miR148aT upon several passages by qPCR. Data is shown in arbitrary units (AU) as the mean ± SEM of four independent experiments, calculated as the 2^ΔCt^ between the miRNA-targeted viruses and Ad-wt. Differences between slopes were analyzed using *F*-test for nonlinear models *** p<0.001.

We have recently shown that miR-148a can selectively control adenovirus activity when the 3′UTR of the E1A gene was genetically engineered with miRNA binding sites recognizing miR-148a [8]. Here, we generated a miR-148a targeted adenovirus regulating the L5 gene by insertion of 8 copies of perfect complementarity for miR-148a downstream the stop codon generating a new regulatory 3′UTR and designated as Ad-L5-8miR148aT (Fig. 1A). MIA PaCa-2 cells stably expressing miR-148a (MIA PaCa-2 miR-148a), or a seed-scrambled miR-148a sequence (MIA PaCa-2 miR-SC) (Supplementary Table S1) were transduced with Ad-wt or Ad-L5-8miR148aT; and the L5 protein product fiber was analyzed by western blot 72 hours later. Similar levels of fiber expression were observed in MIA PaCa-2 miR-SC transduced with either virus. However, a significant reduction in fiber content was detected in MIA PaCa-2 miR-148a that received Ad-L5-8miR148aT, suggesting a miR-148a control of fiber expression. No downregulation of fiber expression was observed in RWP-1 and PANC-1 pancreatic cancer cells that do not express miR-148a (Fig. 1C, Supplementary Table S1). Analysis of the E1A gene expression revealed a downregulation in E1A levels in miR-148a expressing cells at 72h after infection but not at 24h (Fig. 1B). The reduction in E1A at 72h in miR-148a positive cells could be the consequence of decreased viral particles production in Ad-L5-8miR148aT infected cultures. To test this hypothesis, we first analyzed the virus progeny in cellular supernatants of MIA PaCa-2 miR-148a and MIA PaCa-2 miR-SC infected with either Ad-wt or Ad-L5-8miR148aT. Both viral infective particles and viral genomes from MIA PaCa-2 miR-148a cultures infected with Ad-L5-8miR148aT were significantly lower than those treated with Ad-wt whereas no differences between the two viruses were detected in MIA PaCa-2 miR-SC infected cells (Fig. 2A, 2B).

The replication of the adenoviral genome is an event mediated by the viral DNA polymerase (E2B) that takes place earlier than the transcription of late proteins. With this view, we hypothesized that in the first round of infection the viral genomes within miR-148a rich cells would not be substantially different between Ad-wt and Ad-L5-8miR148aT since the miRNA control takes place at a late structural protein whereas, the assembly and release of viral particles would be highly impaired. The comparative analysis of intracellular and extracellular viral genomes between Ad-wt and Ad-L5-8miR148aT in miR-148a positive or negative cells revealed a reduction of viral particles only in the supernatant of miR-148a positive cells (Fig. 2C). The impairment of Ad-L5-8miR148aT to release viral particles in miR-148a cells was maximized when cells were exposed to consecutive rounds of infection (Fig. 2D). Similar reduction in the viral release was observed with the E1A miR-148a controlled adenovirus Ad-E1A-miR148aT (Fig. 2D).

### Ad-L5-8miR148aT ameliorates adenoviral-induced liver toxicity following systemic delivery

**Figure 3:**
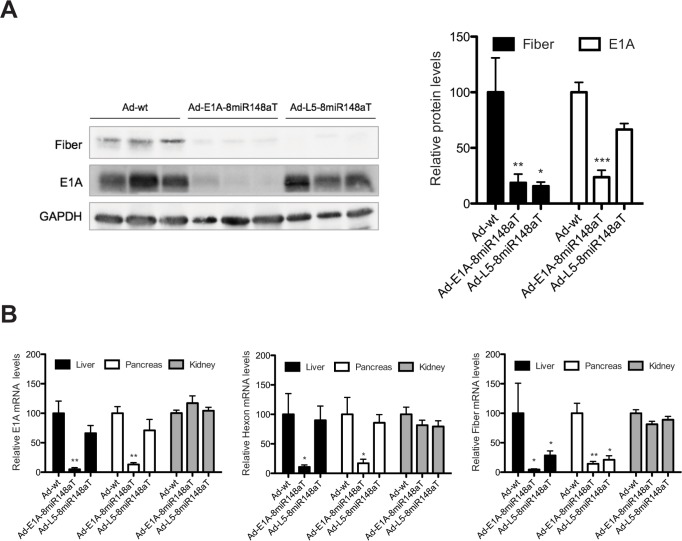
Fiber expression is directly or indirectly regulated, in miR-148a positive tissues, following Ad-L5-8miR148aT or Ad-E1A-8miR148aT systemic delivery (A). Representative Western blots of Fiber in liver extracts from wild type mice intravenously injected with 2×10^10^ vp/mice of Ad-wt, Ad-E1A-8miR148aT and Ad-L5-8miR148aT. Quantification of Fiber signal normalized to GAPDH expression (n=10). Values are expressed relative to Fiber content from Ad-wt treated mice. * and ** denote p<0.05 and p<0.01, respectively. (B). Relative expression of E1A, Hexon and Fiber compared to Ad-wt assessed by RT-qPCR in liver, pancreas and kidneys (n=10/treatment). * and ** denote p<0.05 and p<0.01, respectively.

Intravenous delivery of adenovirus triggers liver-associated toxicity since the liver sequesters most of the adenoviral particles. We sought to evaluate the liver detargeting capacity of Ad-L5-8miR148aT and compared it to that of Ad-E1A-8miR148aT and Ad-wt. Viruses were injected into the tail vein at 2×10^10^ vp/mL and three days later mice were euthanized and fiber expression was analyzed in liver extracts. Fiber protein was detected in all mice injected with Ad-wt, whereas negligible expression was observed from both Ad-L5-8miR148aT and Ad-E1A-8miR148aT (Fig. 3A). A tendency to E1A reduced expression was observed in Ad-L5-8miR148aT whereas a strong decrease was detected in the Ad-E1A-8miR148aT treated mice (Fig. 3A). To further evaluate the detargeting effects of miR-148a-regulated viruses, we analyzed the mRNA content of the early gene E1A, the L3 (hexon) and L5 (fiber) gene expression in liver and pancreas, two organs in which miR-148a is expressed, and in kidney where there is no expression (Supplementary Table S2). E1A mRNA was reduced in liver and pancreas of Ad-E1A-8miR148aT treated mice when compared to Ad-wt, a tendency of reduced E1A mRNA levels was observed in Ad-L5-8miR148aT infected mice. In line with the lack of regulation in kidney, no differences in E1A expression levels between the three viruses were observed in this organ. Similar data was obtained when we analyzed hexon mRNA expression. In contrast, the content of fiber mRNA was extremely reduced in the liver and pancreas of mice treated with both Ad-L5-8miR148aT and Ad-E1A-8miR148aT as compared to Ad-wt. Again, no differences between the different viruses were observed in kidney (Fig. 3B).

**Figure 4:**
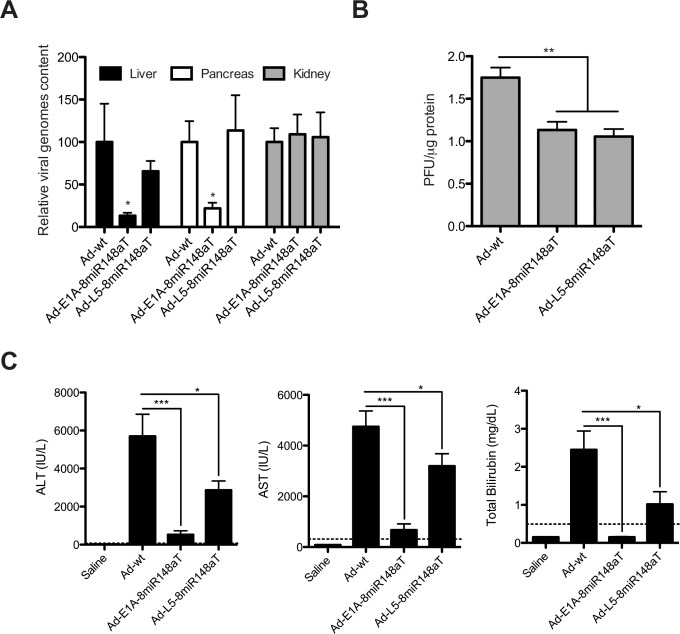
Ad-L5-8miR148aT replication is attenuated in mice liver and displays reduced hepatotoxicity following systemic delivery A viral dose of 2×10^10^vp of Ad-wt, Ad-E1A-8miR148aT or Ad-L5-8miR148aT was intravenously delivered to wild-type mice C57BL/6 mice (n=10). Four days later liver and blood samples were collected. (A). Relative viral replication compared to Ad-wt assessed by genomic qPCR of the L3 gene in liver, pancreas and kidneys (n=10/treatment). * p<0.05. (B). Viral production from livers of mice treated with Ad-wt, Ad-E1A-8miR148aT and Ad-L5-8miR148aT assessed by hexon immunostaining (n=10/treatment). ** p<0.01. (C). Assessment of hepatotoxicity by the determination of AST, ALT and total bilirubin in the serum. Dashed lines correspond to the reference values for C57BL/6 mice. *, ** and *** denote p<0.05, p<0.01 and p<0.001, respectively.

Despite the impairment of human Ad5 to productively replicate in mice, certain level of replication has been reported in the mouse liver [7]. Moreover, we have recently shown that miR-148a prevents from liver and pancreas replication to Ad-E1A-8miR148aT but not to Ad-wt. We investigated whether miR-148a could attenuate Ad-L5-8miR148aT viral replication. The analysis of viral genomes in liver and pancreas was similar to Ad-wt. Reduced viral genomes were only detected in Ad-E1A-8miR148aT treated mice, in miR-148a positive tissues (Fig. 4A). Interestingly the analysis of infective particles produced from livers treated with Ad-E1A-8miR148aT and Ad-L5-8miR148aT was significantly reduced when compared to Ad-wt, suggesting an effective miR-148a control of Ad-L5-8miR148aT *in vivo* (Fig. 4B).

We next assessed the toxicity associated to a single intravenous administration of Ad-L5-8miR148aT and compared to that of Ad-wt and Ad-E1A-8miR148aT. Viruses were injected at a dose of 2×10^10^ vp/mouse and three days later animals were killed. Liver function was evaluated by measuring transaminases and total bilirubin in the sera. In the Ad-wt group there were significant elevations in AST, ALT and total bilirubin levels, which were substantially lower for the Ad-E1A-8miR148aT group. Interestingly, mice treated with Ad-L5-8miR148aT showed significant less induction of ALT, AST and bilirubin indicative of attenuated viral toxicity (Fig. 4C).

### Ad-L5-8miR148aT engineered adenovirus shows similar cytotoxicity to Ad-wt and Ad-E1A-8miR-148aT and triggers strong antitumor activity

**Figure 5:**
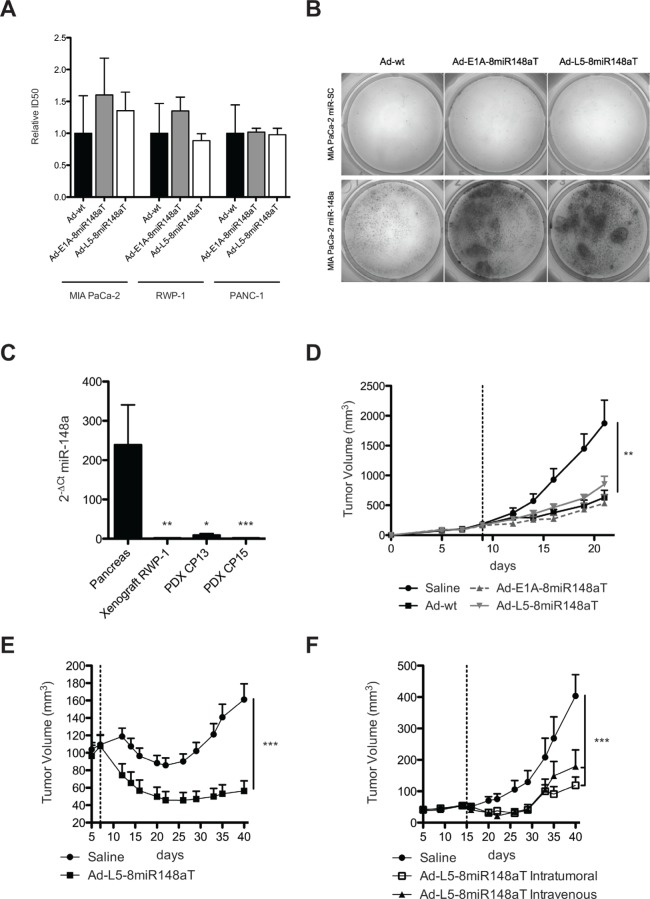
Ad-L5-8miR148aT shows oncolytic potency *in vitro* and strong antitumoral activity in RWP-1 xenografts and PDX models (A). Cytotoxicity assays in the indicated cell lines. Half growth inhibitory concentration (IC50) was calculated for each cell line from dose-response curves. Data is shown as the mean ± SEM of four independent experiments. (B). Cytotoxic effects of Ad-wt, Ad-E1A-8miR148aT and Ad-L5-8miR148aT supernatants obtained after 7 consecutives passages of amplification in MIA PaCa-2 miR-148a and MIA PaCa-2 miR-SC cells. Representative image obtained by methylene blue staining of the culture. (C). RT-qPCR expression of miR-148a from non-tumor human pancreas (n=11) and from RWP-1 xenograft (n=4) and CP13 (n=4), CP15 (n=4) PDX. (D). Follow-up of tumor volumes. RWP-1 xenografts were treated intratumorally with a single injection of 5×10^10^vp/tumor Ad-L5-8miR148aT (n=8), Ad-E1A-8miR148aT (n=8) and Ad-wt (n=8) or with saline (n=8). ** p<0.01. E. Follow-up of tumor volumes. CP13 PDX were treated intratumorally with a single injection of 5×10^10^vp/tumor Ad-L5-8miR148aT (n=10) or with saline (n=10). *** p<0.001. F. Follow-up of tumor volumes. CP15 PDX were treated intratumorally (n=12) or intravenously (n=10) with a single injection of 5×10^10^vp/tumor Ad-L5-8miR148aT or with saline (n=10). *** p<0.001.

To assess the antitumor activity of Ad-L5-8miR148aT, we first performed cytotoxicity assays in the pancreatic cancer cells RWP-1, PANC-1 and MIA PaCa-2 and compared to Ad-E1A-8miR-148aT and Ad-wt activity. Similar ID50 values were obtained with all three viruses in each of the cell lines, illustrating no interference from the engineered sequences in the lytic activity of Ad-L5-8miR148aT (Fig. 5A). Nevertheless cytotoxicity was highly impaired in the miR-148a positive cells (MIA PaCa-2 miR-148a), showing miR-148a dependency of the Ad-L5-8miR148aT lytic activity (Fig. 5B). Next, we analyzed the antitumor efficacy of Ad-L5-8miR148aT in three tumor models with loss of miR-148a expression: xenografts from RWP-1 cells and PDXs from patients CP13 and CP15 (Fig. 5C). RWP-1 subcutaneous tumors were intratumorally injected with a single dose of 5×10^10^ vp/tumor of Ad-L5-8miR148aT, Ad-E1A-8miR-148aT or Ad-wt. Treatment with the different viruses produced a significant inhibition of tumor growth that was similar for the three viruses (Fig. 5D). CP13 PDX subcutaneous tumors treated with a single dose of 5×10^10^ vp/tumor of Ad-L5-8miR148aT showed a significant inhibition in tumor growth and reduced tumor weight (Fig. 5E, Supplementary Fig. S1A) 35 days after treatment. Ad-L5-8miR148aT also induced a significant tumor growth delay and reduced tumor weight in CP15 PDX when administered either intratumorally (i.t.) or systemically (i.v.) (Fig. 5F and Supplementary Fig. S1B). The tumor size of Ad-L5-8miR148aT-treated groups was 2.3-fold, 2.9-fold, 3.4-fold, and 2.3-fold smaller compared to non-treated tumors in RWP-1, CP13, CP15 i.t. and CP15 i.v. models, respectively. In agreement with the *in vitro* cytotoxicity miRNA-regulated viruses did not compromise their *in vivo* antitumor effects and Ad-L5-8miR148aT showed strong antitumor activity in three tumor models.

## DISCUSSION

Oncolytic adenoviral treatment for localized pancreatic tumors can be approached by retrograde intraductal adenoviral administration [10]. However, advanced-stage pancreatic cancer is considered a systemic disease. Consequently, treatments based on oncolytic adenoviruses will require intravenous delivery. Under these circumstances it will be necessary to circumvent undesired associated toxicities mostly related to the significant adenoviral liver uptake. Thus, strategies to develop oncolytic adenovirus with liver attenuation activity are highly desired. In the current work, we propose the downstream L5 region in the adenoviral genome, as a novel region for developing tumor-specific conditionally replicating adenovirus, by exploiting tissue-specific miRNA regulation.

L5 is the last of five cassettes of transcripts (L1-L5) in a large mRNA transcript processed into five individual mRNA families via alternative splicing and usage of different poly(A) signals [9]. The expression of the late mRNAs is controlled by the major late promoter. The complexity of the MLP transcriptional regulation has moved away investigators from studying tumor-specific promoter regulation of late genes to confer oncoselectivity and no separate regulation of the different late mRNAs has been assessed until now. Our data shows it is feasible to achieve oncoselectivity based on tumor deregulated miRNAs by inserting miRNA binding sites in the 3′UTR of a late gene (L5). We observed a robust suppression of the fiber protein (L5 protein product) in cells expressing miR-148a infected with Ad-L5-8miR148aT. Consequently, viral particle release and viral infectivity were reduced. This decrease in the new-formed viral particles led to a reduction in the E1A protein at 72h due to a second round of infection. Noticeably, after 7-rounds of infection the amount of viral particles in the supernatants of infected miR-148a positive cells was almost negligible, most probably as a result of accumulative reduction of viral particles in each replication cycle. In line with high miR-148a expression in the mouse liver, we observed reduced fiber protein and mRNA content in livers of mice receiving Ad-L5-8miR148aT. Consequently, the release of infected particles was highly impaired. This is consistent with the experiments carried out with fiberless adenovirus showing that the fiber protein is absolutely necessary for the production of fully infectious and correctly assembled particles [11]. Furthermore, low abundance of the fiber protein has been associated to low efficiency of adenoviral encapsidation [12, 13].

The infectious particles released from liver extracts had similar infectivity either if they were coming from Ad-L5-8miR148aT or Ad-E1A-8miR148aT treated animals. In contrast, we only observed a reduction in viral genomes in the livers of mice receiving Ad-E1A-8miR148aT but not in those treated with Ad-L5-8miR148aT, in line with the *in vitro* data showing only reduced viral genomes in the cellular supernatant but not intracellularly in cultures infected with Ad-L5-8miR148aT. This is not surprising since late mRNAs are processed after adenoviral genome replication. Thus, the reduced fiber content will interfere with viral particle formation. However, the similarity on viral release from both viruses suggest that miRNA control of structural or early-regulatory proteins display equivalent susceptibility of triggering strong impairment in liver viral production at long term, after multiple replication cycles. It is worth to note that the observed liver detargeting effects will not only account from miR-148a binding to the inserted target sites but also from the recognition by all the miR-148/152 family members, since miR148a, miR-148b and miR-152 can recognize miR-148a target sites and downregulate transgenes with engineered target sites [8].

The improvement in the hepatotoxicity of Ad-L5-8miR148aT was limited, in contrast to that achieved with the Ad-E1A-8miR148aT. This is consistent with the virus control in a late protein. In mice the toxicity peak at 3-4 days following viral administration is the result of the expression of viral proteins, immediately after viral capture, and particularly E1A expression has been reported to be the main cause of toxicity [14]. In this line, we have shown that on day 3 livers infected with Ad-L5-8miR148aT show reduced fiber expression but slight indirect modulation on E1A content. However, this toxicity will most probably transient since there would be minimal successive infections due to the impairment in viral release of Ad-L5-8miR148aT infective particles. Studies performed in swine, which allows productive replication of human Ad5, have shown that the liver toxicity produced by the intravenous administration of high doses of Ad5 presents an initial peak 24 hours after viral administration that drops down until a greater toxicity peak that appears at day 7 as a consequence of the viral replication dynamics [15]. Nevertheless, decreasing the translational capacity of E1A will be desired to complement liver-specific miRNA-mediated fiber suppression. Combined strategies controlling E1A either with tumor-specific promoters together with miRNA-engineered L5 may provide oncolytic adenoviruses with a safer profile and effective antitumor activity. An interesting feature of the current approach is the feasibility to achieve independent control of late genes, as shown by the strong downregulation of the fiber mRNA (L5) but not of the hexon mRNA (L3) after Ad-L5-8miR148aT infection of miR-148a positive tissues. This opens the possibility to similarly regulate additional structural proteins thus increasing the number of sites in the adenoviral genome susceptible to be engineered for oncoselectivity.

Nowadays, these strategies might be even more effective for tumor targeting after systemic delivery than the incorporation of mutations in the Ad5 hexon protein to abolish binding of the capsid to coagulation factor X [16, 17]. Although, such approach reduces viral sequestration and improves adenoviral liver detargeting, factor X recruitment by Ad5 acts as a defense mechanism against complement, therefore, FX ablated adenovirus would be exposed to increased attack by complement and result in suboptimal transduction of target tissues [18].

In this study we have focused on miR-148a, however L5-miRNA targeting could be applied to other miRNAs, such as miR-122, let-7 or miR-199, which have also shown great success in excluding gene expression from the hepatocytes in E1A-miRNA adenoviruses [5, 7, 19-21]. However, an interesting aspect of using miR-148a is that it has been proposed as one of the three miRNAs (together with miR-217 and miR-375) which downregulation is a signature of PDAC [22]. The downregulation of miR-148a initiates in early stages of pancreatic tumorigenesis, in the preneoplasic lesions PanIN-1B, -2 and -3 [23, 24]. Thus, lost of miR-148a in PDAC is a highly frequent and early event. Therefore this will assure the antitumor activity of miR-148a engineered adenoviruses in PDAC, in line with the strong antitumor effects triggered by Ad-L5-8miR148aT in RWP-1 xenografts and in CP13 and CP15 PDX models.

Therefore, these results demonstrate that engineering adenovirus with 8-miR148aT binding sites downstream the L5 adenoviral gene contributes to overcome some limitations of systemic oncolytic adenoviral delivery, such as hepatotoxicity without compromising the antitumor efficacy. Noticeable, these data provides the first example of engineering the L5 (fiber) gene to attenuate viral activity in non-tumoral tissues.

## METHODS

### Cells lines and tissue specimens

The pancreatic cell lines MIA PaCa-2, PANC-1, and HEK293 were obtained from the American Type Culture Collection (ATCC). RWP-1 cells were derived from human pancreatic adenocarcinoma biopsies perpetuated as xenograft in nude mice [25]. MIA PaCa-2 miR-148a and MIA PaCa-2 miR-SC cells were obtained in the laboratory as previously described [8]. All cells lines were maintained in Dulbecco's modified Eagle's medium supplemented with 10% fetal bovine serum (Gibco BRL, Carlsbad, CA).

Human pancreas was obtained from patients diagnosed of pancreatic ductal adenocarcinoma in Hospital Clinic of Barcelona who underwent surgical resection. All specimens were obtained according to the Institutional Review Board-approved procedures for consent. Informed consent was obtained from all subjects and all the experiments conformed to the principles set out in the WMA Declaration of Helsinki. Mouse pancreas, liver and kidney were obtained from C57BL/6 adult mice.

### Generation of adenovirus controlled by miRNA target sites

Ad-E1A-8miR148aT has been previously described [8]. Ad-wt was obtained from the ATCC (Manasas, VA).

Ad-L5-8miR148aT genome was generated by the following steps: First the pXK3.1 plasmid, which contains the wild type fiber was mutated in the PolyA sequence and the overlapping STOP codon with the primer 5′-CACTTTTTCATACATTGCCCAAGAATGACTCGA GAATCGTTTGTGTTATGTTTCAACG-3′ to generate the sequence GAATGACTCGAGAA, containing a XhoI restriction site using the QuickChange Multi Site-Directed Mutagenesis kit (Stratagene, Wilmington, NC). The 3′UTR of pEndK-8miR148aT [8] containing the 8 binding sites for miR-148a was amplified using primers Fw 5′-CTCGAGCCCTTTATTAAACTTACATC-3′ and Rv 5′-CTCGAGTGATTGCGTGTGTGGTTAAC-3′ flanked with XhoI restriction site sequences and cloned into the XhoI site of the pXK3.1 mutated plasmid to generate pXK3.1-fiber-8miR148aT.

pAdeno-L5-8miR148aT was generated by homologous recombination of the pXK3.1-fiber-8miR148aT with the genome of the serotype 5 wild-type adenovirus (Ad-wt) in E. Coli BJ5183 cells (Stratagene, Wilmington, NC) as described in [26]. The pAdeno-L5-8miR148aT was transfected to HEK293 cells to obtain viral particles. All viruses were propagated in A549 cells and purified by cesium chloride banding. The concentration of physical viral particles (vp/mL) was determined by means of optical density and that of infectious particles (pfu/mL) by hexon immunostaining [27] in HEK293 cells. All viruses presented similar vp/pfu ratio.

### Western blot analysis

Total protein extracts were obtained with lysis buffer (50 mM Tris-HCl pH 6.8, 2% SDS) containing 1% Complete Mini Protease Inhibitor (Roche Diagnostics GmbH, Basel, Switzerland). Protein concentration was determined by BCA Protein Assay Kit (Pierce-Thermo Fisher Scientific, Waltham, MA) and 35 μg of total protein was resolved by electrophoresis on 7.5% gels and transferred to nitrocellulose membranes by standard methods. Membranes were immunoblotted with a rabbit anti–Adenovirus-2/5 E1A polyclonal antibody (1:200; clone 13 S-5; Santa Cruz Biotechnology, Dallas, TX) or Adenovirus Fiber [4D2] antibody (1:200; GeneTex, San Antonio, TX) 1h at room temperature (RT). Then the blots were rinsed with TBS-T and incubated for 45 minutes at RT with either HRP-conjugated goat anti-rabbit IgG or rabbit anti-mouse IgG antibodies (DakoCytomation, Glostrup, Denmark). Antibody labeling was detected by the enhanced chemiluminescent method (Amersham Biosciences, Amersham, UK). Western blot expression data was normalized to GAPDH (1:10000; Millipore, Billerica, MA).

### Viral progeny production

Total cell lysates from MIA PaCa-2 miR-SC/miR-148a and liver homogenates were obtained at 72 hours post-infection and progeny production was assessed by titration in HEK293 cells by hexon immunostaining. Viral progeny production from several passages was assessed in the supernatant of MIA PaCa-2 miR-SC/miR-148a cells infected with Ad-L5-8miR148aT, Ad-E1A-8miR148aT and Ad-wt. When the cytopathic effect was observed, supernatants were harvested and used to infect new wells of MIA PaCa-2 miR-SC/miR-148a cultures. This was performed for 7 passages and at each passage viral genome quantification was performed.

### Viral genome quantification

Viral DNA was obtained from supernatants, cellular extracts or frozen tissues using the UltraClean BloodSpin DNA Isolation Kit (Mo Bio Laboratories, Carlsbad, CA) according to the manufacturer's instructions. Viral genomes were determined by Real-Time qPCR (100 ng of DNA) using SYBR Green I Master plus mix (Roche Diagnostics, Basel, Switzerland) and the hexon primer sequences: Fw 5′-GCCGCAGTGGTCTTACATGCACATC-3′ and Rv 5′-CAGCACGCCGCGGATGTCAAAG-3′. The adenovirus copy number was quantified with a standard curve, consisting of adenovirus DNA dilutions (10^2^-10^7^ copies) in a background of genomic DNA.

### *In vitro* cell survival studies

Dose–response curves were constructed for MIA PaCa-2 miR-148a, MIA PaCa-2 miR-SC, PANC-1, MIA PaCa-2 and RWP-1. Cells were transduced with doses ranging from 0.001 vp/cell to 10000 vp/cell of Ad-wt, Ad-E1A-8miR148aT or Ad-L5-8miR148aT. Cell viability was measured by a colorimetric assay (MTT Ultrapure, USB Corporation, Cleveland, OH) 3 days post-infection.

### cDNA synthesis and real-time quantitative PCR

RNA was obtained and isolated using miRNeasy Mini Kit (Qiagen, Venlo, The Netherlands). A total of one microgram was reverse transcribed using Moloney Murine Leukemia Virus reverse transcriptase and oligo(dT) (Ambion). One microliter of the reaction was used as a template for the qPCR amplification reaction (LightCycler 480SYBR Green I Master Mix, Roche) in a thermocycler (ViiA 7 Real-Time PCR system, Applied Biosystems). E1A: Fw 5′-CGGCCATTTCTTCGGTAATA-3′, and Rev 5′-CCTCCGGTGATAATGACAAG-3′. Hexon: Fw 5′-GTCTACTTCGTCTTCGTTGTC-3′, and Rv 5′-TGGCTTCCACGTACTTTG-3′. Fiber: Fw 5′-CTCCAACTGTGCCTTTTC-3′, and Rv 5′-GGCTCACAGTGGTTACATT-3′. Quantitative expression data were normalized to Gdx: Fw 5′-GGCAGCTGATCTCCAAAGTCCTGG-3′, and Rv 5′-AACGTTCGATGTCATCCAGTGTTA-3′.

### Quantitative miRNA RT-PCR

Total RNA was obtained from cell cultures or tissues using miRNeasy Mini RNA Extraction Kit (Qiagen). A total of 10 nanograms total RNA were reverse transcribed using a reverse transcriptase and stem-loop primers as indicated by the manufacturer (TaqMan MicroRNA Reverse Transcription Kit – Applied Biosystems). One and a half microliters of the reaction was used as a template for the qPCR amplification reaction (TaqMan Universal Master Mix, No AmpErase UNG – Applied Biosystems) in a thermocycler (ViiA 7 Real-Time PCR system). Quantitative miRNA RT-PCR expression data was normalized to small nucleolar RNA U6 expression (RNU6B). Stem-loop primers and qPCR probes were purchased from TaqMan MicroRNA assay (Applied Biosystems): RNU6B (AB ID: 001093), hsa-miR-148a (AB ID: 000470).

### Toxicity analysis

Blood samples were collected by intracardiac puncture under anesthesia. Serum AST, ALT and total bilirubin were determined on an Olympus AU400 Analyzer (Olympus, Tokyo, Japan).

### Mouse xenografts

RWP-1 cells (2.5×10^6^), embedded in Matrigel 1:1 (BD Biosciences, San Jose, CA), were injected subcutaneously into each flank of male, 6-8-weeks-old, athymic nu/nu mice (Harlan, Sant Feliu de Codines, Spain). PDAC fragments of 10-15 mg from CP15 and CP13 patient-derived xenografts (PDX), patient specimens maintained as xenografts in the mouse pancreas [28, 29] were implanted in the subcutaneous tissue of 7-weeks-old, athymic nu/nu mice.

Viral treatment was administered at day 9-(RWP-1), 7-(CP13) and 15-(CP15) post implantation when tumors achieved a median volume of 170, 100 and 80 mm^3^ respectively. Tumors were measured at least three times a week and their volume was calculated by the formula V = larger diameter × (smaller diameter)^2^ × pi ÷ 6. At the end of the experiment tumor weight was calculated for CP13 and CP15 PDX. All animal procedures met the guidelines of European Community Directive 86/609/EEC, and approved by the local ethical committee.

### Statistical analysis

Data are represented by the mean ± SEM, or population distribution with median and interquartile range (dot plot comparing grouped data). If not specified, statistical differences were evaluated using non-parametric, Mann-Whitney test; or using univariant general linear models with Tukey's b post-hoc test. For all statistical analysis, the level of significance was set as p<0.05. The software used for statistical analysis was IBM SPSS Statistics 20 (IBM, Armonk, NY); data was represented using GraphPad Prism v5.0a (GraphPad Software, La Jolla, CA).

## SUPPLEMENTARY MATERIAL FIGURE AND TABLES



## References

[1] Yamamoto M, Curiel DT (2010). Current issues and future directions of oncolytic adenoviruses. Mol Ther.

[2] Huntzinger E, Izaurralde E (2011). Gene silencing by microRNAs: contributions of translational repression and mRNA decay. Nat Rev Genet.

[3] Spizzo R, Nicoloso MS, Croce CM, Calin GA (2009). SnapShot: MicroRNAs in Cancer. Cell.

[4] Kelly EJ, Russell SJ (2009). MicroRNAs and the regulation of vector tropism. Mol Ther.

[5] Ylosmaki E, Hakkarainen T, Hemminki A, Visakorpi T, Andino R, Saksela K (2008). Generation of a conditionally replicating adenovirus based on targeted destruction of E1A mRNA by a cell type-specific MicroRNA. J Virol.

[6] Girard M, Jacquemin E, Munnich A, Lyonnet S, Henrion-Caude A (2008). miR-122, a paradigm for the role of microRNAs in the liver. J Hepatol.

[7] Cawood R, Chen HH, Carroll F, Bazan-Peregrino M, van Rooijen N, Seymour LW (2009). Use of tissue-specific microRNA to control pathology of wild-type adenovirus without attenuation of its ability to kill cancer cells. PLoS Pathog.

[8] Bofill-De Ros X, Gironella M, Fillat C (2014). MiR-148a- and miR-216a-regulated Oncolytic Adenoviruses Targeting Pancreatic Tumors Attenuate Tissue Damage Without Perturbation of miRNA Activity. Mol Ther.

[9] Young CS (2003). The structure and function of the adenovirus major late promoter. Curr Top Microbiol Immunol.

[10] Jose A, Sobrevals L, Miguel Camacho-Sanchez J, Huch M, Andreu N, Ayuso E, Navarro P, Alemany R, Fillat C (2013). Intraductal delivery of adenoviruses targets pancreatic tumors in transgenic Ela-myc mice and orthotopic xenografts. Oncotarget.

[11] Legrand V, Spehner D, Schlesinger Y, Settelen N, Pavirani A, Mehtali M (1999). Fiberless recombinant adenoviruses: virus maturation and infectivity in the absence of fiber. J Virol.

[12] Henning P, Lundgren E, Carlsson M, Frykholm K, Johannisson J, Magnusson MK, Tang E, Franqueville L, Hong SS, Lindholm L, Boulanger P (2006). Adenovirus type 5 fiber knob domain has a critical role in fiber protein synthesis and encapsidation. J Gen Virol.

[13] van Beusechem VW, van Rijswijk AL, van Es HH, Haisma HJ, Pinedo HM, Gerritsen WR (2000). Recombinant adenovirus vectors with knobless fibers for targeted gene transfer. Gene Ther.

[14] Engler H, Machemer T, Philopena J, Wen SF, Quijano E, Ramachandra M, Tsai V, Ralston R (2004). Acute hepatotoxicity of oncolytic adenoviruses in mouse models is associated with expression of wild-type E1a and induction of TNF-alpha. Virology.

[15] Jogler C, Hoffmann D, Theegarten D, Grunwald T, Uberla K, Wildner O (2006). Replication properties of human adenovirus *in vivo* and in cultures of primary cells from different animal species. J Virol.

[16] Alba R, Bradshaw AC, Parker AL, Bhella D, Waddington SN, Nicklin SA, van Rooijen N, Custers J, Goudsmit J, Barouch DH, McVey JH, Baker AH (2009). Identification of coagulation factor (F)X binding sites on the adenovirus serotype 5 hexon: effect of mutagenesis on FX interactions and gene transfer. Blood.

[17] Waddington SN, McVey JH, Bhella D, Parker AL, Barker K, Atoda H, Pink R, Buckley SM, Greig JA, Denby L, Custers J, Morita T, Francischetti IM, Monteiro RQ, Barouch DH, van Rooijen N (2008). Adenovirus serotype 5 hexon mediates liver gene transfer. Cell.

[18] Xu Z, Qiu Q, Tian J, Smith JS, Conenello GM, Morita T, Byrnes AP (2013). Coagulation factor X shields adenovirus type 5 from attack by natural antibodies and complement. Nat Med.

[19] Callegari E, Elamin BK, D'Abundo L, Falzoni S, Donvito G, Moshiri F, Milazzo M, Altavilla G, Giacomelli L, Fornari F, Hemminki A, Di Virgilio F, Gramantieri L, Negrini M, Sabbioni S (2013). Anti-tumor activity of a miR-199-dependent oncolytic adenovirus. PLoS One.

[20] Ylosmaki E, Lavilla-Alonso S, Jaamaa S, Vaha-Koskela M, af Hallstrom T, Hemminki A, Arola J, Makisalo H, Saksela K (2013). MicroRNA-mediated suppression of oncolytic adenovirus replication in human liver. PLoS One.

[21] Jin H, Lv S, Yang J, Wang X, Hu H, Su C, Zhou C, Li J, Huang Y, Li L, Liu X, Wu M, Qian Q (2011). Use of microRNA Let-7 to control the replication specificity of oncolytic adenovirus in hepatocellular carcinoma cells. PLoS One.

[22] Frampton AE, Giovannetti E, Jamieson NB, Krell J, Gall TM, Stebbing J, Jiao LR, Castellano L (2014). A microRNA meta-signature for pancreatic ductal adenocarcinoma. Expert Rev Mol Diagn.

[23] Hanoun N, Delpu Y, Suriawinata AA, Bournet B, Bureau C, Selves J, Tsongalis GJ, Dufresne M, Buscail L, Cordelier P, Torrisani J (2010). The silencing of microRNA 148a production by DNA hypermethylation is an early event in pancreatic carcinogenesis. Clin Chem.

[24] Xue Y, Abou Tayoun AN, Abo KM, Pipas JM, Gordon SR, Gardner TB, Barth RJ, Suriawinata AA, Tsongalis GJ (2013). MicroRNAs as diagnostic markers for pancreatic ductal adenocarcinoma and its precursor, pancreatic intraepithelial neoplasm. Cancer Genet.

[25] Dexter DL, Matook GM, Meitner PA, Bogaars HA, Jolly GA, Turner MD, Calabresi P (1982). Establishment and characterization of two human pancreatic cancer cell lines tumorigenic in athymic mice. Cancer Res.

[26] Huch M, Gros A, Jose A, Gonzalez JR, Alemany R, Fillat C (2009). Urokinase-type plasminogen activator receptor transcriptionally controlled adenoviruses eradicate pancreatic tumors and liver metastasis in mouse models. Neoplasia.

[27] Cascante A, Abate-Daga D, Garcia-Rodriguez L, Gonzalez JR, Alemany R, Fillat C (2007). GCV modulates the antitumoural efficacy of a replicative adenovirus expressing the Tat8-TK as a late gene in a pancreatic tumour model. Gene Ther.

[28] Perez-Torras S, Vidal-Pla A, Miquel R, Almendro V, Fernandez-Cruz L, Navarro S, Maurel J, Carbo N, Gascon P, Mazo A (2011). Characterization of human pancreatic orthotopic tumor xenografts suitable for drug screening. Cell Oncol (Dordr).

[29] Sobrevals L, Mato-Berciano A, Urtasun N, Mazo A, Fillat C (2014). uPAR-controlled oncolytic adenoviruses eliminate cancer stem cells in human pancreatic tumors. Stem Cell Res.

