# Supplementary material for: Late-phase miRNA-controlled oncolytic adenovirus for selective killing of cancer cells
Source: Oncotarget. 2015 Jan 31;6(8):6179–90. doi: 10.18632/oncotarget.3350 (PMC4467430; doi:10.18632/oncotarget.3350)
Supplement: Supplementary file 1 [file oncotarget-06-6179-s001.pdf]

Late-phase miRNA-controlled oncolytic adenovirus for selective killing of cancer cells

Supplementary Material

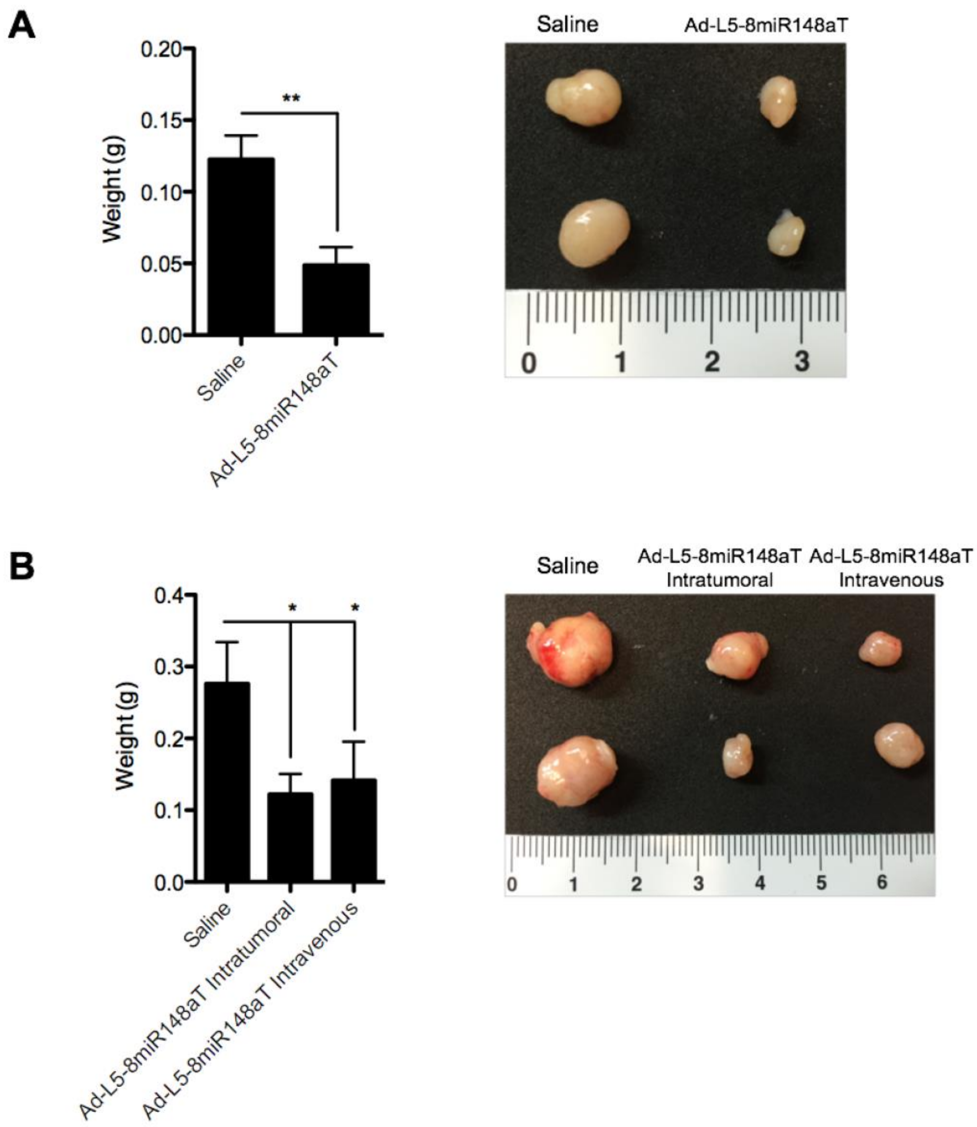

Supplementary Figure S1: **Antitumor efficacy of Ad-L5-8miR148aT**. A) Tumor weight and representative tumor images 35 days post-intratumoral treatment of CP13 PDX. B) Tumor weight and representative tumor images at 25 days post-intratumoral or intravenous treatment of CP15 PDX.

Table S1: miR-148a cellular content

Cell Lines	$2^{\Delta Ct}$ miR-148a MEAN	SEM
MIAPaCa-2 miR-148a	9,87	1,76
MIA PaCa-2 miR-SC	0,83	0,35
MIA PaCa-2	1,03	0,74
PANC-1	1,58	0,37
RWP-1	1,60	1,05

Table S2: miR-148a tissue content

Mouse tissues	$2^{\Delta Ct}$ miR-148a MEAN	SEM
Pancreas	143,00	22,52
Liver	52,92	10,02
Kidney	2,82	0,48
